# Kinetic exchange models of societies and economies

**DOI:** 10.1098/rsta.2021.0170

**Published:** 2022-05-30

**Authors:** Giuseppe Toscani, Parongama Sen, Soumyajyoti Biswas

**Affiliations:** ^1^ Department of Mathematics ‘F. Casorati’, University of Pavia, Pavia, Italy; ^2^ Department of Physics, University of Calcutta, Kolkata, India; ^3^ Department of Physics, SRM University - AP, India; ^4^ Institute for Applied Mathematics and Information Technologies (IMATI), Via Ferrata, 5/A Pavia, Italy

**Keywords:** kinetic theory, wealth exchange, opinion formation, ranking, disease spreading

## Abstract

The statistical nature of collective human behaviour in a society is a topic of broad current interest. From formation of consensus through exchange of ideas, distributing wealth through exchanges of money, traffic flows, growth of cities to spread of infectious diseases, the application range of such collective responses cuts across multiple disciplines. Kinetic models have been an elegant and powerful tool to explain such collective phenomena in a myriad of human interaction-based problems, where an energy consideration for dynamics is generally inaccessible. Nonetheless, in this age of Big Data, seeking empirical regularities emerging out of collective responses is a prominent and essential approach, much like the empirical thermodynamic principles preceding quantitative foundations of statistical mechanics. In this introductory article of the theme issue, we will provide an overview of the field of applications of kinetic theories in different socio-economic contexts and its recent boosting topics. Moreover, we will put the contributions to the theme issue in an appropriate perspective.

This article is part of the theme issue ‘Kinetic exchange models of societies and economies’.

## 1. Introduction

The statistical nature of collective human behaviour in a society is a topic of broad current interest.

Historically, however, physics models of society pre-date Newtonian mechanics. First Thomas Hobbes (1588–1679) and then Sir William Petty (1623–1687) (founding member and vice-president of the Royal Society) argued for the use of physics laws and their quantitative reproducibility in social sciences (see [1] for a review of historical developments).

The term ‘social physics’ was coined by Auguste Comte in the 1830s [2]. Maxwell himself, before starting his works on distribution of velocities of atoms in a gas, was influenced by the works of Henry Buckle [3], who argued about emergent ‘order, symmetry and law’ in a society. Boltzmann too compared gas particles to individuals in a society: ‘The molecules are like so many individuals, having the most various states of motion…’ [4].

Therefore, it is rather apt that a renewed surge of interest in physics models of society and economies came through kinetic exchange models seeking to reproduce empirical laws by relying on the averaging out of the details of human interaction complexities by laws of large numbers. In 1931, Meghnad Saha & B. N. Srivastava noted the similarity between colliding atoms and market interactions [5]. This was subsequently highlighted by Mandelbrot in 1960 [6]. Years later, Angle proposed an elementary version of the kinetic exchange model for society [7].

The kinetic models of wealth exchange, considering interactions of individuals as random events with conservation of money, led to rather robust representation of Pareto’s universal power law distribution of wealth, highlighting its underlying inequality. The power law, however, in keeping with kinetic theory of gases, does not represent a critical phenomenon. On the other hand, in formation of opinion in a society, no conservation law exists. It was then possible for the first time to obtain a symmetry breaking transition to consensus from fragmented opinions, belonging to the Ising universality class.

The kinetic rule of new elementary interactions, with conserving or non-conserving output, has since been an elegant and powerful tool to explain the formation of universal profiles in collective phenomena in a myriad of human interaction-based problems, where an energy consideration for dynamics is generally inaccessible [8]. Nonetheless, in this age of Big Data, seeking empirical regularities emerging out of collective responses is a prominent and essential approach, much like the empirical thermodynamic principles preceding quantitative foundations of statistical mechanics.

In this theme issue, we intend to bring together the current progress in the applications of kinetic exchange models in various phenomena associated with societies (opinion formations, rating, social networks, fake news, etc.) and economies (inequality measures, taxation, trade models, behavioural economics, etc.) using numerical simulations, machine learning techniques, analytical methods and data analysis, reported by physicists, social scientists, mathematicians and economists. In the following, we summarize the individual contributions of this issue, put in four broad categories.

## 2. Kinetic models of wealth distribution

A considerable part of the contributions to this issue are related to aspects of wealth distribution. The models are based on exchange of goods and money in binary interactions, with several different approaches to the details of the modelling, all leading to different macroscopic models and stationary distributions. In these binary exchanges, like in the case of kinetic theory of gases, there is generally an underlying conservation principle applied for the total wealth.

Bisi proposes a kinetic model to describe trade transactions between agents who live in different nations, and who may migrate from one country to another [9]. The transactions depend on each population’s propensity to trade and on random effects. On the kinetic level, described by a system of Boltzmann-type equations, a continuous exchange limit is considered, leading to a system of Fokker–Planck type equations, with specific contributions that account for the rate of transfer.

In Boghosian *et al.* [10], the properties of a three-parameter model of asset exchange, the Affine Wealth Model, are studied in relation to some important theories of twentieth-century neoclassical economics, such as Expected Utility Theory, General Equilibrium Theory, and Prospect Theory. The result of this analysis is that the phenomenology exhibited by the Affine Wealth Model is fundamentally incompatible with Expected Utility Theory and General Equilibrium Theory, but very similar to that exhibited by Prospect Theory. Based on these observations, the research leads us to conclude that Prospect Theory provides, at least for the topic of wealth distribution, a close connection between econophysics and neoclassical economics.

Paul *et al.* study the steady-state characteristics of the income distribution and the resulting inequality measures (Gini and Kolkata indices) in kinetic models based on two different types of binary transactions [11]. In the first case, the exchange takes place between randomly chosen pairs of agents and in the next, one of the agents in the chosen pair is the poorest of all and the other agent is taken at random from the rest of the population. In addition, different values of the saving propensity are considered. Numerical results allow the inequality indices to be related to the types of exchange, and to the value of the saving propensity coefficient.

Goswami [12] studies the dynamics of agents below a threshold line in a kinetic wealth exchange model derived from the model by Chatterjee, Chakrabarti and Manna (CCM model) where a distribution of saving propensities was assumed for individuals [13]. These agents are eligible for subsidy as can be seen in any real economy. An interaction is prohibited if both of the interacting agents’ wealth falls below the threshold line. A walk for such agents can be conceived in the abstract Gain–Loss Space (GLS) and is macroscopically compared to a lazy walk. The effect of giving subsidy once to such agents is checked over giving repeated subsidy from the point of view of the walk in GLS. It is seen that the walk has more positive drift if the subsidy is given once. The correlations and other interesting quantities are studied.

Stojkoski *et al.* [14] explore the role of non-ergodicity in the relationship between income inequality, the extent of concentration in the income distribution, and income mobility, the feasibility of an individual changing their position in the income rankings. Fitting the model to empirical data for the income share of the top earners in the USA, they find evidence that the income dynamics are consistently in a regime in which non-ergodicity characterizes inequality and immobility.

Neñer *et al.* [15] study genetic machine learning algorithms to train agents in the Yard-Sale model. The main result indicates that for more significant fraction of rational agents, the inequality at the collective level becomes greater.

Ludwig & Yakovenko [16] present an interesting survey on the approaches to economic inequality based on ideas from statistical physics and kinetic theory. The origins of the exponential Boltzmann–Gibbs distribution and the Pareto power law are discussed in relation to additive and multiplicative stochastic processes. A second part of the paper analyses income distribution data in the USA for the time period 1983–2018 using a two-class decomposition. Interestingly, the growth pattern of the income inequality have been subsequently shown to follow some universal trends seen in markets with unrestricted competitions [17].

## 3. Kinetic exchange models of opinion formation

In the cases where individuals in a society interact by exchanging opinions, there is no underlying conservation principle unlike the cases of wealth exchange. Therefore, such interactions can lead to formation of consensus or a state of fragmented opinions, depending on the nature of the exchanges. The nature of this ‘transition’ is reminiscent of that seen in magnetic systems with ordered (finite net magnetization) and disordered (zero net magnetization) phases.

The notion of realizing a walk in a virtual space inspired by a stochastic model for social dynamics, as considered in [12], was also used by Saha & Sen [18] for a kinetic exchange model of opinion dynamics [19]. In this work, the opinion changes of an individual due to binary exchanges mentioned above were mapped to virtual random walks with or without memory. The walk can be biased or unbiased depending upon the nature of the interaction between the agents, which can either lead to a state of consensus or that of fragmented state of opinions. Some of the features of the walks have been argued to be comparable to the critical quantities associated with the mean field Ising model, to which class this opinion dynamics model belongs.

Roy & Biswas [20] have studied the dynamics of opinion formation in a society where the internally held beliefs and externally expressed opinions of the individuals, which are not necessarily the same at all times, were taken into account. This was done using a kinetic exchange opinion model, similar to the ones mentioned above. The difference between these two quantities can play a significant role in assessing public opinion and consequently lead to changes in policies, which are not necessarily reflective of the individual’s internally held beliefs.

Pires & Crokidakis [21] study a model of opinion dynamics considering activation/deactivation of agents. In other words, individuals can become inactive and drop out from the discussion. A probability governs the deactivation dynamics, whereas social interactions are ruled by kinetic exchanges, considering competitive positive/negative interactions. Inactive agents can become active due to interactions with active agents. The analytical and numerical results showed the existence of two distinct non-equilibrium phase transitions, with the occurrence of three phases, namely ordered (ferromagnetic-like), disordered (paramagnetic-like) and absorbing phases. The absorbing phase represents a collective state where all agents are inactive, i.e. they do not participate in the dynamics, inducing a frozen state.

During & Wright [22], motivated by recent successes in model based pre-election polling, proposed a kinetic model for opinion formation which includes voter demographics and socio-economic factors like age, sex, ethnicity, education level, income and other measurable factors like behaviour in previous elections or referenda as a key driver in the opinion formation dynamics. The model is based on Toscani’s kinetic opinion formation model [23] and the leader–follower model of Düring *et al.* [24], and leads to a system of coupled Boltzmann-type equations and associated, approximate Fokker–Planck type systems. Numerical examples using data from general elections in the UK show the effect different demographics have on the opinion formation process and the outcome of elections.

Loy *et al.* [25] propose a Boltzmann-type kinetic description of opinion formation on social networks, which takes into account a general connectivity distribution of the individuals. The opinion exchange processes considered here were inspired by the Sznajd model and related simplifications but the individuals are not placed on a regular lattice, nor it is the case of mean field interaction as was the case for few of the above-mentioned studies. Instead, the structure of a social network was followed statistically, where the contacts of a given individual determine their influence. They find a polarization switching under certain conditions.

## 4. Kinetic models of ranking

An interesting application of collisional kinetic theory is presented in [26]. Düring *et al.* study a refined kinetic version of the Elo rating system, a model originally proposed by Arpad Elo for chess [27], nowadays considered to be one of the most important rating systems in sports, economics and gaming. A kinetic version of the Elo model was proposed by Junca and Jabin in 2015 [28]. Here, the model by Junca and Jabin is generalized to account for variable performance of individual players or teams, and the consequent dynamics are illustrated with computational results.

## 5. Kinetic models in compartmental epidemiology

The spread of the COVID-19 pandemic has led to the development of compartmental epidemiology models, in which statistical mechanics methods applied to multi-agent systems have been the hallmark.

In this issue, Bellomo *et al.* present, in a multiscale framework, a unified approach to modelling the COVID-19 pandemic, from contagion to within-host dynamics [29]. The model focuses on both vaccination and therapeutic actions, and also takes into account the action of vaccination plans related to the emergence of new variants. A detailed presentation, starting from modelling and followed by a qualitative analysis, is proposed, and well-focused simulations enlighten the achievements of the theoretical analysis.

A control problem of the epidemic spreading has been studied by Dimarco *et al.* [30], with the aim of understanding and limiting the effects of closure strategies, measures that have resulted in severe economic consequences. Starting from a recent kinetic model that takes into account the heterogeneity described by the social contact of individuals [31], the effects of an optimal control strategy whose action is to selectively limit the average number of contacts and consequently reduce the number of infected cases in the population have been evaluated. Using a data-driven approach, it is shown that the model helps to evaluate the effects of social constraints.

In Franceschi & Pareschi’s contribution [32], compartmental epidemiology models are used to study the propagation of fake news. Indeed, the rise of social networks as a primary means of communication has simultaneously triggered an increase in the amount of fake news circulating online. The urgent need for models that can describe the growing infodemic of fake news has been highlighted by the COVID-19 pandemic. Misinformation has caused vaccination campaigns to slow down and generally increased the inability of individuals to discern the reliability of information. Using the powerful tools of kinetic theory, Franceschi & Pareschi describe the interaction between the diffusion of fake news and the competence of individuals through compartmental models in which fake news spreads like an infectious disease with different impact depending on the level of competence of individuals.

## Data Availability

All data used in this work are publicly available.
